# Adaptation of Gut Microbiome to Transgenic Pigs Secreting β-Glucanase, Xylanase, and Phytase

**DOI:** 10.3389/fgene.2021.631071

**Published:** 2021-03-04

**Authors:** Jianxin Mo, Guoling Li, Guangyan Huang, Haoqiang Wang, Junsong Shi, Rong Zhou, Gengyuan Cai, Zhenfang Wu, Xianwei Zhang

**Affiliations:** ^1^National Engineering Research Center for Breeding Swine Industry, Wens Foodstuff Group Co., Ltd., Yunfu, China; ^2^College of Animal Science, South China Agricultural University, Guangzhou, China

**Keywords:** transgenic pigs, microbial enzymes, gut microbiome, metagenomics, feed efficiency

## Abstract

We previously generated transgenic pigs with enhanced growth rate and reduced nutrient loss. However, the composition of their gut microbiome is unknown. In this study, we successfully generated *EGFP* marker-free transgenic (MF-TG) pigs with high expression levels of microbial β-glucanase, xylanase, and phytase in the parotid gland. We collected intestinal contents from the ileum, cecum and colon of five MF-TG and five wild-type (WT) sows and investigated the gut microbiome of the transgenic pigs via metagenomic analysis. Results showed that the levels of probiotics, such as *Lactobacillus reuteri* and *Streptococcus*, were more abundant in the cecum of the MF-TG pigs and higher than those of WT pigs. By contrast, the levels of harmful microorganisms, such as *Campylobacter*, *Chlamydia trachomatis*, and *Campylobacter fetus*, and various unidentified viruses, were higher in the cecum of the WT pigs than those of the MF-TG pigs. By comparing unigenes and the eggNOG database, we found that the microorganisms in the colon of the MF-TG pigs had high fractional abundance in DNA (cytosine-5)-methyltransferase 1 and serine-type D-Ala-D-Ala carboxypeptidase, whereas the aspartate carbamoyltransferase regulatory subunit and outer membrane protein pathways were enriched in the WT pigs. Moreover, the microorganisms in the cecum of the MF-TG pigs were active in GlycosylTransferase Family 8 (GT8), Glycoside Hydrolase Family 13 (GH13), and Glycoside Hydrolase Family 32 (GH32). Furthermore, the levels of numerous carbohydrases, such as glucan 1,3-beta-glucosidase, xylan 1,4-beta-xylosidase and exo-1,3-1,4-glucanase, were higher in the cecum of the MF-TG pigs than those of the WT pigs. The results indicated that intestinal microbes can change adaptively to the secretion of transgenic enzymes, thereby forming a benign cooperation with their host. This cooperation could be beneficial for improving feed efficiency.

## Introduction

Livestock industries pursue maximum animal growth rate to utilize the full genetic potential of animals. However, anti-nutrient factors, such as non-starch polysaccharides (NSPs) and phytates, adversely affect feed efficiency, resulting in inefficient feed digestion and substantial rates of nutrient leaching into the environment (Xianwei et al., 2018). Pigs produce considerable amounts of nitrogen (N) and phosphorus (P) because they are inherently incapable of digesting NSPs and phytates, which are present in feed grain (Golovan et al., 2001). Various methods have been developed to improve pig nutrient utilization (Swiatkiewicz et al., 2016). Dietary supplements with phytate- or NSP-degrading enzymes can effectively reduce P and N emissions and improve feed utilization. However, the effects of supplements are limited by feed production processes, enzyme activity stability and cost. Genetically engineered pigs expressing phytate- or NSP-degrading enzymes in their salivary glands offer an alternative and useful strategy for dietary supplementation. Golovan et al. (2001) reported that transgenic (TG) pigs that produce salivary phytase can remarkably improve P digestion from soybean meals. Several other researchers also successfully generated these types of TG pigs (Lin et al., 2015). In our previous studies, we successfully generated TG pigs expressing three microbial enzymes, namely, β-glucanase, xylanase, and phytase, in their salivary glands. These enzymes considerably enhanced the digestion of NSPs and phytates in feedstuff (Xianwei et al., 2018). However, these TG pigs also systemically express the enhanced green fluorescent protein (EGFP) maker, which may have negative effects on pig health and food safety (Bi et al., 2016).

The mammalian gut microbiome plays critical roles in normal digestive functions, nutrient utilization, antibiotic resistance, and defense against pathogens (Sommer and Bäckhed, 2013). Dyspeptic gut microbiota are associated with several intestinal and extraintestinal diseases and poor animal growth performance. These conditions increase the risk of food safety and public health hazards and result in low profitability of animal production (Wang et al., 2019). Therefore, intestinal microbiome and their interactions with animal hosts have long been a notable research interest. Previous studies adopted various animal models, including TG and knock out (KO) animals, to explore the relationship of host genes to the functions of the gut microbiome, but none of them directly focused on feed efficiency (Qingqing et al., 2016). Thus, whether or not the endogenous phytate- or NSP-degrading enzymes (phytase, β-glucanase, and xylanase) of TG pigs would alter the composition and activity of intestinal microbiome remains unanswered. In the present study, we successfully produced *EGFP* marker-free transgenic (MF-TG) pigs by deleting the *EGFP*-coding gene in our previously generated TG pigs via the cyclization recombination enzyme (Cre)–LoxP recombination system. The transgenes were expressed efficiently in the salivary gland and not expressed in other tissues of the MF-TG pigs. We further tested whether or not the MF-TG pigs would affect the functional contributions and biological roles of intestinal microbes via metagenomic analysis.

## Materials and Methods

### Ethics Statement

The protocol implemented in this study was in accordance with the Instructive Notions with Respect to Caring for Laboratory Animals issued by the Ministry of Science and Technology of China. This study was approved by the Animal Care and Use Committee of the South China Agricultural University.

### Deletion of the *EGFP* Marker by Cyclization Recombination Enzyme

The TG pigs we raised in a previous study (Xianwei et al., 2018) were mated with wild-type (WT) sows and then slaughtered on day 30. The fetuses in the womb were removed, and primary pig fetal fibroblasts (PFFs) were isolated by adherence. The *EGFP*-tag was deleted by cyclization recombination enzyme (Excellgen, United States). The PFFs were cultured for another 24 h. Half of the cells were placed into a new 24-well plate. The steps above were repeated twice until the cells grew into a monoclone.

### Generation of Cloned Pigs by Somatic Cell Nuclear Transfer

The marker-free PFFs derived from a single colony were used as nuclear donors for somatic cell nuclear transfer, and the embryos were cultured *in vitro* overnight. Afterward, the embryos were transferred to the oviducts of recipient sows. Antibiotics were injected for four consecutive days to reduce inflammation, and the physiological conditions of the recipient sows were recorded daily. In addition, 1000 IU of pregnant mare serum gonadotropin (PMSG) was injected into the recipient sows on the 10th day after embryo transfer, and 800 IU of human chorionic gonadotrophin (hCG) was injected on the 13th day to maintain pregnancy.

### PCR and Southern Blot Analyses of Founder Pigs

Genomic DNA of founder (F0) cloned pigs were isolated using a DNA tissue kit (OMEGA, United States) in accordance with the manufacturer’s protocol. Primer pairs of P1-F/R, P2-F/R, and P3-F/R were designed to amply the mPSP promoter, bg17-eg1314 dual tansgenes, and marker-free region, respectively. TG and KO genes were amplified via PCR. The PCR condition and procedure were set in accordance with the manufacture’s protocol of PrimeSTAR Max DNA Polymarase (TaKaRa, Japan). The PCR products were analyzed by 1.5% agarose gel. For Southern blot, probe 1 and probe 2 were designed to target bg17-eg1314 dual transgenes and marker-free region, respectively. A total of 20 μg of genomic DNA was digested and then analyzed by 0.8% agarose gel at 30 V for 16 h. Subsequently, the gel was washed with alkaline solution, neutralization solution and 20 × SSC solution and then transferred onto nylon membranes. Genomic DNA was hybridized with digoxigenin-labeled DNA probe. After hybridization, the nylon membranes were washed and detected with a buffer by using a DIG-high prime DNA labeling and detection starter kit II (Roche, Germany) following the manufacturer’s instructions. Finally, the membranes were imaged using UVP software. The sequences of the primers and probes are shown in Supplementary Table 1.

### Enzymatic Activity Assay and Western Blot Analysis

Saliva samples of adult MF-TG pigs were collected using non-fat cotton balls. A portion of the samples was tested for enzymatic activity assay at the optimal pH. Enzymatic activity assays were performed as described in our previous work (Xianwei et al., 2018). In detail, β-glucanase and xylanase activity assays were based on estimating the amount of reducing sugars released from the relevant substrates in the reactions using 3,5-dinitrosalicylic acid (DNS) reagent. One unit of activity was defined as the quantity of enzyme that releases reducing sugar at the rate of 1 mmol/min. Phytase activity was determined by means of vanadium molybdenum yellow spectrophotometry. The reaction was performed in a final volume of 600 mL solution containing 0.25 M acetate buffer (pH 5.5), 5 mM sodium phytate, and 50 mL enzyme preparation at 39°C for 30 min, followed by termination of the reaction by adding 400 mL of an ammonium molybdate-ammonium vanadate-nitric acid mixture. After mixing and centrifugation, the absorbance was measured at a wavelength of 415 nm. One unit of phytase activity was defined as the amount of activity that liberates one micromole of phosphate per minute at 39°C.

The other portion of the saliva samples was centrifuged using Amicon Ultra-15 (Millipore, United States) for Western blot detection. In brief, a total of 20 μg of protein was subjected to sodium dodecyl sulfate (SDS) polyacrylamide gel electrophoresis and then transferred onto PVDF membranes (Millipore, United States). The membranes were blocked with 5% non-fat dry milk for 2 h and then incubated overnight at 4°C with the primary antibodies of HA (Hemagglutinin) protein tag (ab137838, Abcam) or GAPDH (ab8245, Abcam). The membranes were thoroughly washed and then further incubated with a secondary antibody for 2 h at room temperature. Finally, the membranes were imaged using UVP software.

### Recording of Production Performance

The MF-TG pigs (F0) were crossed with WT Duroc sows, and F1 generation MF-TG pigs were propagated and grown until they reached 35 kg in weight (about 130 days of age). A total of 14 MF-TG boars (36.93 ± 4.77 kg) and 11 WT littermate boars (39.6 ± 9.57 kg) were divided into two measuring stations. Ten MF-TG gilts (34.71 ± 6.06 kg) and 10 WT littermate gilts (37.88 ± 5.97 kg) were allocated to three pens according to their initial weight; among them, six MF-TG gilts and eight WT littermate gilts were raised in individual pens. All pigs were allowed free access to water and fed the same experimental diet formulations made in accordance with the Nutrient Requirements of Swine (NRC, 2012) (Supplementary Table 4). The experimental formula was designed with 2% protein reduction, no mineral P additives, and low energy level to evaluate the functional efficiency of the MF-TG pigs. During the experiment, production performance was recorded using MK3 Fire feeders (Fire, United States). In addition, a total of 11 WT boars and 11 gilts were separately tested and fed complete formula feed as the WT2 (Supplementary Table 11). Fresh dung samples of the pigs were randomly collected every morning toward the end of the last 5 days of the trial. The dung samples were stored in a refrigerator at 4°C. Finally, the samples were mixed well and dried at 80°C. Measurement of growth performance was ended when the body weight of the pigs reached 115 kg (about 220 days of age).

### Dietary Treatment Experiments

Fresh pig manure was collected at the time of flushing the hog house (08:00 and 17:00) for five consecutive days. Multipoint collection was adopted with consistent sampling quantity of each point. Manure samples were frozen immediately at −20°C. Afterward, the manure of each group was uniformly and thoroughly mixed. Sampling was performed through the quartile method. Total nitrogen was measured using fresh dung samples. The remaining samples were dried at 80°C. The samples were air-dried and then crushed to particles <0.425 mm in size for Ca and P content measurements.

### Metagenomics Sequencing and Statistical Analyses

Five MF-TG sows (115.76 ± 1.57 kg) and five WT sows (116.04 ± 0.55 kg) were selected for metagenomic analysis. During the fattening stage, the pigs were raised with the same experimental diet under human-controlled farm conditions and similar management schemes. The intestinal contents were collected from the ileum, cecum and colon and then immediately transferred to liquid nitrogen for temporary storage. The genomic DNA of the samples was extracted using Magnetic Soil and Stool DNA Kit (TIANGEN^®^, China) in accordance with the manufacturer’s protocol, and used to construct the sequencing library. The NEBNex^*R*^ UtraTM DNA Library Prep Kit for Illumina (NEB, United States) was used to prepare the DNA library. The Raw data was obtained from the Illumina HiSeq sequencing platform. Clean data was generated via removal of low-quality reads in raw data. The specific processing steps are as follows: (a) remove the reads which contain low quality bases (default quality threshold value ≤38) above a certain portion (default length of 40 bp); (b) remove the reads in which the N base has reached a certain percentage (default length of 10 bp); and (c) remove reads which shared the overlap above a certain portion with Adapter (default length of 15 bp).

Clean data was blast to the pig genome database as default using Bowtie2.2.4 software to filter the reads that are of host origin. The parameters are as follows: -end-to-end, -sensitive, -I 200, -X 400. The sequences of the transgenes were also removed from the sequencing data.

The sequencing data was assembled by single sample assembly and mixed assembly. For the single-sample assembly, the Clean Data was assembled and analyzed by using SOAPdenovo (V2.04) software. The parameters are as follows: -d 1, -M 3, -R, -u, -F, -K 55. Then, the assembled Scaftigs were interrupted from N connection and left the Scaftigs without N. All Clean Data of the samples were compared to each Scaffolds by using Bowtie2.2.4 software to acquire the PE reads not used. The parameters are as follows: -end-to-end, -sensitive, -I 200, -X 400. All reads not used in the single assembly of all samples were combined. SOAPdenovo (V2.04) software was used to conduct the mixed assembly under the same parameters used in the single assembly. Then, the fragments shorter than 500 bp were filtered in all of Scaftigs for statistical analysis of data generated from the single or mixed assembly.

Scaftigs (≥500 bp) assembled from single and mixed samples were used to predict the open reading frames (ORFs) by using the MetaGeneMark software. ORFs with length shorter than 100 nt were filtered from the predicted results with default parameters. For ORF prediction, CD-HIT software was used to remove redundancies and obtain unique initial gene catalogs. Clean data of each sample were mapped onto an initial gene catalogs by using Bowtie2.2.4. The number of reads to which genes mapped in each sample was obtained under the following parameters: end-to-end, sensitive, I 200 and X 400. In each sample, the gene with ≤2 reads was filtered to obtain the gene catalog (unigenes) for subsequent analysis. On the basis of the number of mapped reads and gene lengths, the abundance information of each gene in each sample was analyzed.

DIAMOND software was used to BLAST the unigenes to the sequences of bacteria, fungi, archaea, and viruses, which were extracted from the NR database of NCBI with the parameter settings blastp-e 1e-5. For the aligned results of each sequence, the result with the e value ≤ the smallest e value × 10 was selected, considering that each sequence may have multiple aligned results. The LCA algorithm was applied to the system classification of MEGAN software to ensure the species annotation information of the sequences. A table containing the number of genes and the abundance information of each sample in each taxonomic hierarchy was constructed on the basis of LCA annotation results and the gene abundance table. LEfSe analysis was performed to determine differences in species composition among groups. Permutation test between groups was used for the Metastats analysis of each taxonomic group and to obtain the *P* value. The Benjamini and Hochberg False Discovery Rate were utilized to correct the *P* value and acquire the *q* value. LEfSe analysis was conducted by using LEfSe software. Finally, random forest was implemented to construct a random forest model. Important species were screened by Mean Decrease Accuracy and Mean Decrease Gin.

DIAMOND software was used to BLAST the unigenes to the functional database with the parameter settings blastp, -e 1e-5. The functional databases included the KEGG, eggNOG, and carbohydrate enzyme (CAZy). For each sequence’s BLAST result, the best BLAST Hit was utilized for subsequent analysis. The relative abundance of different functional hierarchies was analyzed. In this study, the relative abundance of each functional hierarchy was equal to the sum of relative abundance annotated to that functional level. On the basis of functional annotation results and the gene abundance table, the gene number table of each sample in each taxonomic hierarchy was obtained. The gene number of a function in a sample was equal to the gene number annotated to this function and the abundance was non-zero.

For resistance gene annotation, resistance gene identifier (RGI) software was employed to align the unigenes to the CARD database (version 2.0.1) under the parameter settings blastp, e value ≤ 1e-30. The relative abundance of ARO was counted from the aligned results. On the basis of the abundance of ARO, abundance bar charts and abundance cluster heat maps were created, and differences in the number of resistance genes between groups were determined. Furthermore, analyses of the abundance distribution of the resistance genes in each sample, species attribution of resistance genes and resistance mechanism of resistance genes were conducted via comparing the gene catalog and CARD database.

### Statistical Analyses

Data was analyzed by using the GLM procedure (SAS, United States). For growth performance, covariance analysis was performed, and initial body weight and experimental period were used as the covariates. For apparent fecal nutrient emission, one-way ANOVA followed by Duncan’s multiple comparison were conducted. Unpaired two-sample *t*-test (two-tailed test) was used for enzymatic activity analyses. Statistical significance was set to *P* < 0.05.

## Results

### Generation of MF-TG Pigs

**FIGURE 1:**
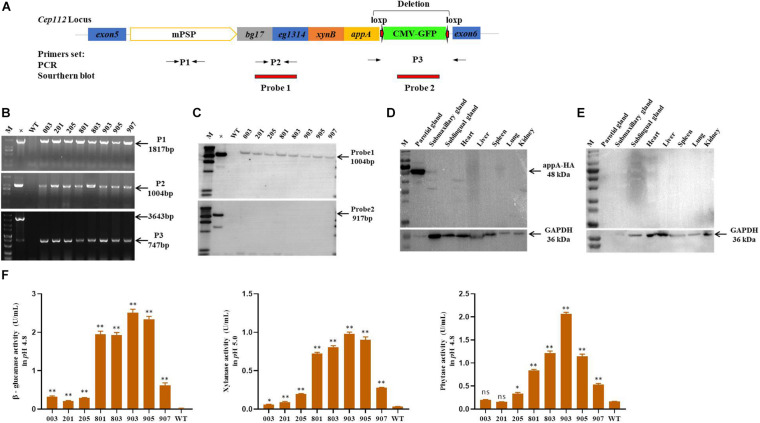
Characterization of transgene and expression in founder MF-TG pigs. **(A)** Four microbial enzymes were integrated into porcine *CEP112* intron 5 after deleting the *EGFP*-tag. Different primers (P1, P2, and P3) were used to confirm the occurrence of transgene, Probe 1 and probe 2 were designed for Southern blot analysis. **(B)** Genomic DNA of the cloned pigs was amplified by PCR and analyzed by gel electrophoresis. **(C)** Southern blot analysis revealed that multiple enzyme transgenes were integrated into porcine *CEP112* intron 5 without the *EGFP* maker. **(D)** Western blot analysis demonstrated that the microbial enzymes were specifically expressed in the parotid gland of the MF-TG pigs. **(E)** Western blot analysis demonstrated that microbial enzymes were not detected in the WT pigs. **(F)** MF-TG pigs could efficiently express β-glucanase, xylanase, and phytase in their salivary gland. Values are shown as mean ± SEM. ^∗^*P* < 0.05, ^∗∗^*P* < 0.01 versus control.

The *EGFP*-tag was deleted by the Cre-Loxp recombination system in the porcine fetal fibroblasts derived from a single colony, and the MF-TG pigs were generated by somatic cell nuclear transfer technique (SCNT) to remove the potential effect of EGFP protein on the gut microbiome. A total of 2001 reconstructed embryos were transferred to the oviduct of eight recipient sows and four sows became pregnant successfully. Seventeen alive piglets and two stillborn piglets were born (Supplementary Table 13). Eight piglets grown healthily and were selected for subsequent experiments. The primers of PCR and Southern blot were used to identify TG fragments (Figure 1A). Results of PCR and Southern blot showed that all of the eight pigs were MF-TG pigs (Figures 1B,C). Different tissues and organs of the MF-TG pigs were collected. Western blot revealed that the MF-TG pigs efficiently expressed β-glucanase, xylanase, and phytase only in the parotid gland and not in other tissues and WT pigs (Figures 1D,E). During the feeding period, we collected saliva samples from 6-month-old MF-TG and WT pigs for enzymatic activity assays. The assays detected enzymatic activity in all MF-TG pigs, among which 903 had the highest enzyme activity; 2.5, 0.98, and 2.07 U/mL of β-glucanase, xylanase, and phytase were detected, respectively (Figure 1F).

### MF-TG Pigs Had Improved Feed Utilization and Reduced Nutrient Emission

**FIGURE 2:**
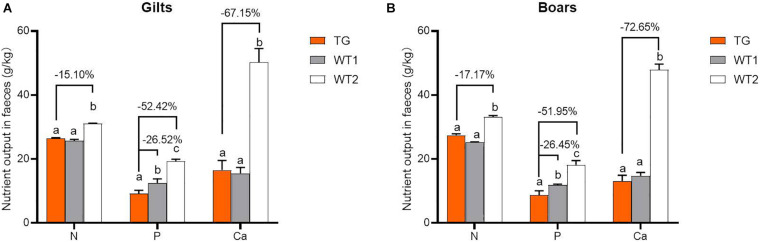
Comparison of nutrient emissions of the MF-TG pigs and WT littermates. Utilization of fecal calcium (Ca), nitrogen (N), and phosphorus (P) was detected in the MF-TG gilts **(A)** and boars **(B)**. Data are shown as mean ± SEM. MF-TG (*n* = 5) and WT1 (*n* = 6) pigs were fed experimental diets with low nitrogen, no mineral phosphorus, and low energy level. WT2 pigs (*n* = 11) were fed commercial diets. Different letters represent *P* < 0.05.

MF-TG boars (803, 903, and 907) were crossed with WT Duroc sows. A total of 48 offspring were born, of which 24 were MF-TG pigs and 24 were WT littermate pigs. A total of 24 MF-TG pigs (14 boars and 10 gilts) and 21 WT littermates (11 boars and 10 gilts) were raised together and fed nutrition-deficient experimental diets to measure the growth performance of the MF-TG pigs (Supplementary Table 4). Although the difference in average daily feed intake (ADFI) between the MF-TG and WT pigs was not significant (*P* = 0.95 and 0.05 for male and female, respectively), the MF-TG pigs had better feed consumption, higher average daily gain (ADG), better feed conversion rate (FCR) and shorter day to market those of the WT pigs. Feed consumption decreased by 11.85–21.57 kg, ADG increased by 131.34–162.88 g, FCR declined by 0.18–0.42, and day to market shortened by 9.42–15.07 days, respectively (Table 1). We further investigated the effects of the microbial enzymes secreted by MF-TG pigs on nutrient emission. Results showed that the P emission of the MF-TG pigs significantly decreased by approximately 26.45–26.52%, but their N and Ca excretion did not significantly change compared with that of the WT1 group. Compared with WT2 boars fed commercial diets for breeding pigs (Supplementary Table 11), the fecal N, P and Ca contents of the MF-TG boars decreased by 17.10, 51.95, and 72.65%, respectively (Figure 2A), and those of the MF-TG gilts declined by 15.10, 52.42, and 67.15%, respectively (Figure 2B). The MF-TG pigs were as good as or even better than the WT2 control group in terms of growth performance (Supplementary Table 12).

**TABLE 1:** Comparison of growth performance between F1 MF-TG and their WT littermates fed experimental diets during the growing period from 30 to 100 kg.

	Male					Female				
		
Items	TG (*n* = 14)	WT (*n* = 11)	Pooled SEM	P-value	Change	TG (*n* = 9)	WT (*n* = 10)	Pooled SEM	*P*-value	Change
ADFI	1.86	1.87	0.06	0.95	–0.01	9.22	7.85	0.44	0.05	1.37
DVISITS	6.62	8.14	0.51	0.0788	–1.52	2.01	1.93	0.06	0.325	
Total feed	122.68	144.25	2.92	0.0002	–21.57	138.92	150.77	2.22	0.0023	–11.85
Consumption, kg										
Days to market, day	65.94	81.01	2.3	0.0013	–15.07	69.67	79.09	1.99	0.0054	–9.42
ADG, g/d	971.11	808.23	35.41	0.009	162.88	948.12	816.78	29.93	0.0085	131.34
FCR	1.94	2.36	0.05	<0.0001	–0.42	2.17	2.35	0.03	0.0025	–0.18

*ADFI, average daily feed intake; DVISITS, feeding frequency; ADG, average daily gain; FCR, feed conversion rate. Pens effect was defined as random effect. Use covariance analysis for statistics, covariates: initial weight and batch, when *P* < 0.01, data was correct by covariates.*

### Comparison of the Gut Microbial Communities of MF-TG and WT Sows

**FIGURE 3:**
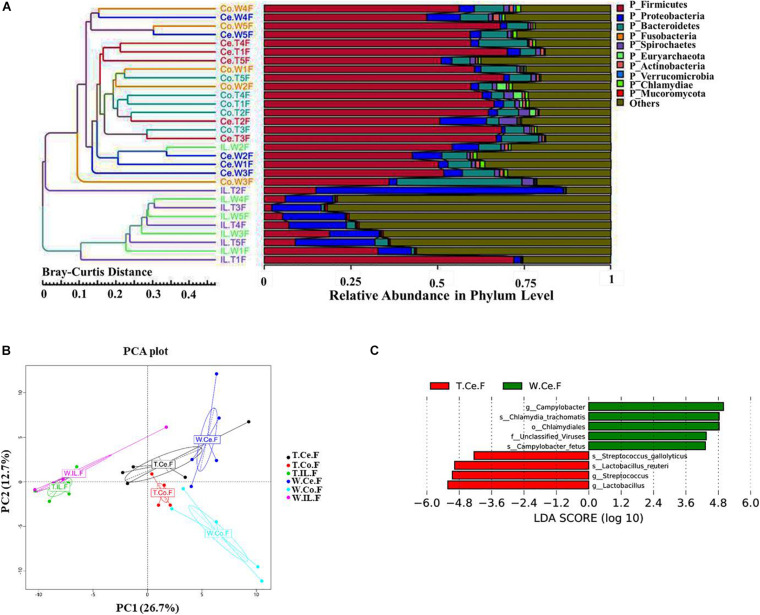
Comparison of intestinal microbial communities of the MF-TG pigs and WT littermates. **(A)** Community composition at the phylum level in MF-TG females and WT littermates. “IL,” “Ce,” and “Co” stand for ileum, cecum, and colon, respectively. MF-TG and WT female pigs stand for TF and WF, respectively. The Bray–Curtis distance was used for cluster analysis of relative abundance at the phylum level to detect similarities in different samples. **(B)** Principal component analysis (PCA) results of the intestinal microbiota at the phylum level. **(C)** LDA value distribution of different species shown as a histogram. Length of the histogram represents the relative effect of different species.

We collected intestinal contents from the ileum, cecum, and colon of five MF-TG sows and five WT sows and investigated their gut microbiome via metagenomic analyses. A total of 1,832,628 predicted genes (71.26%) were annotated into the NR database, and their proportions at the kingdom, phylum, class, order, family, genus, and species levels were 82.85, 79.04, 72.80, 72.25, 60.07, 55.22, and 42.53%, respectively (Supplementary Table 5). We selected the top 10 microorganisms with the largest relative abundance in each sample and integrated the clustering results with the relative abundance at the phylum level by using the Bray–Curtis distance for cluster analysis. At the phylum level, the abundance of ileal (IL) microorganisms was substantially lower than those of cecal (Ce) and colonic (Co) microorganisms. Nevertheless, individual samples were highly variable. The main Ce and Co microorganisms were *Firmicutes*, *Bacteroidetes*, and *Euryarchaeota*, whereas the primary IL microorganisms were *Proteobacteria* and *Firmicutes*. Furthermore, the difference in the microbiome of the same intestinal parts of MF-TG pigs and WT pigs was not significant at the phylum level (Figure 3A and Supplementary Table 6). We also detected no difference in relative abundance at the class, order, family, genus, and species levels of the top10 microorganisms in the same intestinal parts between the MF-TG pigs and WT pigs (data not shown). Principal component analysis (PCA) revealed that the distance between IL and Co/Ce was far and clustered into a different category, but Co and Ce could not be separated (Figure 3B and Supplementary Table 7). We analyzed the differences in species composition among different groups via LEfSe. We evaluated the abundance of different species by using LDA scores. Results showed that the WT pigs had significantly higher proportions of harmful microorganisms, including *Campylobacter*[causes diarrhea (Burnham and Hendrixson, 2018)], *Chlamydia trachomatis* (associated with pneumonia (Lu et al., 2012), and *Campylobacter fetus* [causes septicemia (Sachse and Grossmann, 2002)] and various unidentified viruses, than the MF-TG pigs. By contrast, the MF-TG pigs had higher levels of probiotics, such as *Lactobacillus reuteri* and *Streptococcus*, in cecum than the WT pigs (Figure 3C).

### Comparison of Functions and Abundance of Microbial Genes of MF-TG and WT Pigs

**FIGURE 4:**
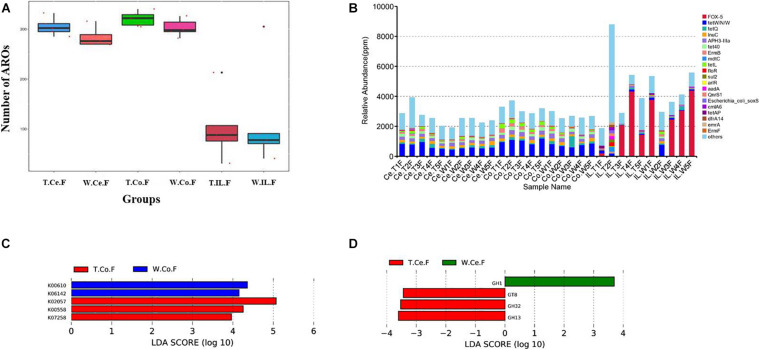
Functional annotation and abundance of microbial genes in different intestinal microorganisms. **(A)** Number of ARO in the MF-TG and WT pigs was not significantly different. **(B)** Relative abundance of the top 20 ARO is shown as a histogram. “Ppm” stand for parts per million. LEfSe analysis revealed the different functions in KEGG **(C)** and CAZy **(D)** databases.

**FIGURE 5:**
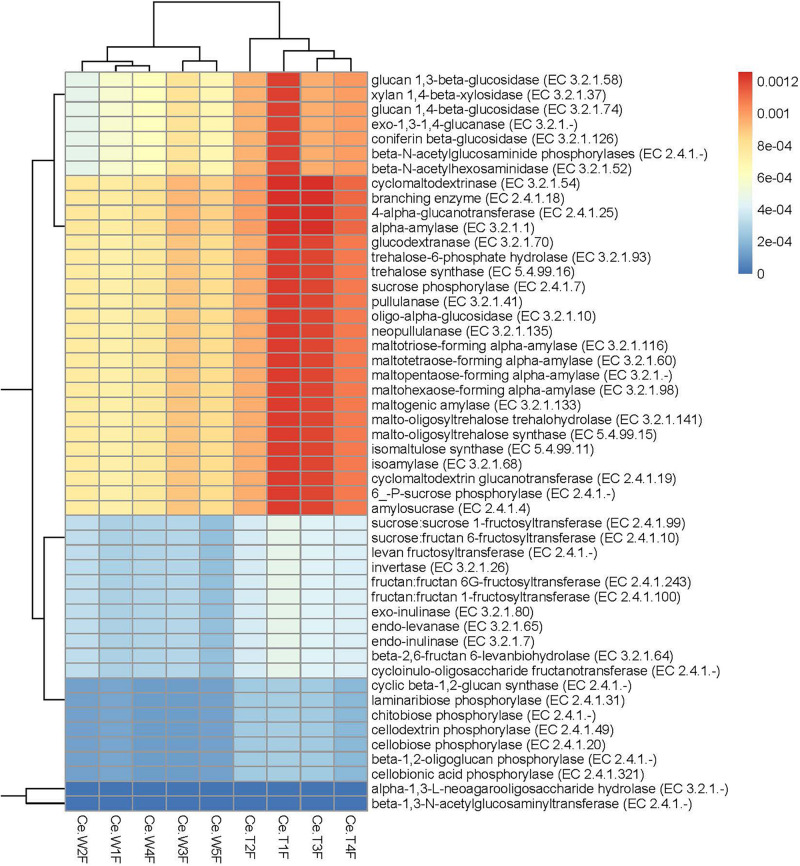
Significantly enriched carbohydrases in the cecum of MF-TG pigs. Color code stands for relative abundance.

For resistance gene annotation, unigenes were compared with the CARD database by using the RGI software. Results showed that the difference in the abundance of resistance genes of the MF-TG and WT pigs was not significant (Figure 4A and Supplementary Table 8). Moreover, common veterinary drugs corresponded with resistance genes, such as tetracycline resistance protein, aminoglycoside antibiotic kinase, lincomycin resistance, tetracycline efflux gene, erythromycin resistance and florfenicol resistance gene, in the pig intestines. The abundance of Fox-5, a cephalosporin resistance gene, was the highest in the ileum, whereas tetW was the highest in the colon and cecum (Figure 4B and Supplementary Table 9). The microorganism carrying resistance genes of common antibiotics may acquire from external environment where these type of microorganism exist pervasively due to long history of antibiotics abuse. We analyzed the different KEGG functions of MF-TG and WT pigs. Results showed that the microorganisms in the colon of the MF-TG pigs had high fractional abundance in DNA-methyltransferase 1 (K00558, cysteine and methionine metabolism) and serine-type D-Ala-D-Ala carboxypeptidase (K07258, peptidoglycan biosynthesis), whereas the aspartate carbamoyltransferase regulatory subunit (K00610, nucleotide metabolism) and outer membrane protein (K06142, signaling and cellular processes) pathways were enriched in the WT pigs (Figure 4C). A comparison of the unigenes and CAZy database revealed that the gene abundance of transgenic cecal microorganisms was active in GT8 (Glycosyl Transferase Family 8), GH13 (Glycoside Hydrolase Family 13), and GH32 (Glycoside Hydrolase Family 32) (Figure 4D). We then analyzed the CAZy database by using DIAMOND software. Results showed that a total of 50 carbohydrate enzymes were significantly enriched in the cecal microorganisms of MF-TG pigs (Figure 5 and Supplementary Table 10). Among them, enzymes involved in the hydrolysis of NSPs, such as glucan 1,3-beta-glucosidase (EC 3.2.1.58), exo-1,3-1,4-glucanase (EC 3.2.1.-), xylan 1,4-β-xylosidase (EC3.2.1.37), and coniferin β-glucosidase (EC3.2.1.126), and starch hydrolases participated in starch and limit dextrins hydrolysis, such as α-amylase (EC3.2.11), oligo-1,6-glucosidase (EC3.2.1.10), and glucan 1,4-α-maltotetraohydrolase (EC3.2.1.60), were most enriched (Figure 5). In addition, the level of levan hydrolases, sucrose hydrolases, trehalose hydrolases and many phosphorylases related to metabolism of carbohydrates were up-regulated. Surprisingly, the relative abundance of some polysaccharide synthases, including 1,4-α-glucan branching enzyme (EC2.4.1.18), 4-α-glucanotransferase (EC2.4.1.25), trehalose synthase (EC5.4.99.16), malto-oligosyltrehalose synthase (EC 5.4.99.15), isomaltulose synthase (EC 5.4.99.11), and cyclic beta-1,2-glucan synthase (EC 2.4.1.-), were also induced.

## Discussion

Generation of genetically modified pigs is an efficient strategy for improving various indicators of pig performance, such as feed utilization, piglet survival rate and pork nutritional composition. In our previous study, we generated transgenic pigs expressing four microbial enzymes, namely, *bg17A*, *eg1314*, *xynB*, and *eappA*, in the salivary glands specifically (Xianwei et al., 2018). During the feeding process, we found that the N and P emissions of the TG pigs substantially reduced, whereas their nutrient intake and absorption from the feed increased. However, the TG pigs carry the *EGFP* gene. This gene has potential animal and human health hazards (Stepanenko et al., 2008). In the present study, we developed TG pigs without the *EGFP* marker. The MF-TG pigs were found to efficiently secrete the microbial enzymes phytase, β-glucanase, and xylanase. The expression levels of the transgenes varied between different MF-TG pigs. Our previous work (Xianwei et al., 2018) showed that the expression levels of the transgenes were high during feeding time and 10 min before and after feeding time and significantly reduced during rest time in the TG pigs. These results indicated that feeding behavior induced the expression of transgenes. In the present study, pigs were given free access to feed. Some pigs showed high gene expression level when the saliva samples were collected during their feeding time and the others showed low gene expression levels when the saliva samples were collected during their rest time. This phenomenon may explain the wide expression levels of the transgenes in the MF-TG pigs. We compared the MF-TG pigs fed nutrition-deficient experimental diets, which contain lower nitrogen level, no mineral phosphorus additive, and lower energy level than commercial diet, with the WT pigs fed commercial diets. Results showed that the genetically modified pigs considerably reduced their N, P, and Ca emissions in the manure without compromising production performance. When compared with the WT pigs fed the same nutrition-deficient experimental diets, these genetically modified pigs had substantially improved ADG and feed utilization efficiency but only reduced fecal P emissions. This result was not consistent with that of our previous trial in metabolic cage under restrictive feeding and movement (Xianwei et al., 2018). The main difference between the previous and the present studies was that all pigs herein were measured in cages without feeding and movement restrictions. Other prior studies reported that supplementary enzymes in the feed have a positive effect on the digestibility of feed nutrients (Swiatkiewicz et al., 2016; Recharla et al., 2019). Phytase can liberate P from phytate by step-wise dephosphorylation of phytate. β-glucanase and xylanase can effectively degrade glucan and xylan, respectively. Hence, β-glucanase, xylanase, and phytase are both nutritionally and ecologically beneficial because they enhance P/N absorption while reducing P/N excretion (Xianwei et al., 2018).

The issue of whether or not the digestive enzymes secreted by genetically modified pigs would affect their intestinal microbiome remains unclear. In recent years, metagenomic methods based on high-throughput sequencing have rapidly promoted the study of the composition and function of intestinal microorganism floras (Costea et al., 2018). In the present study, all experimental pigs were selected from populations with a similar genetic background, of the same gender and raised under the same environmental, nutritional, and management conditions to minimize the variability caused by genetic, gender and external factors. Nevertheless, results showed that the gut microbiomes of the MF-TG and WT pigs were different, consistent with the results of previous studies (Nicholson et al., 2012; Parks et al., 2013; Quan et al., 2019). The MF-TG pigs had higher levels of probiotics, such as *L. reuteri* and *Streptococcus*, in the cecum than the WT pigs. *L. reuteri* strongly adheres to the intestinal mucosa; thus, this bacterium can improve the distribution of intestinal microbes, antagonize the colonization of other harmful bacteria and prevent the development of intestinal diseases (Kleerebezem and Vaughan, 2009). In addition, *L. reuteri* can produce reuterin, a non-protein broad-spectrum antibacterial substance that can greatly inhibit the growth of Gram-positive/negative bacteria, yeast, fungi and pathogens (Kleerebezem and Vaughan, 2009). *Streptococcus* is generally considered a health-promoting microorganism because of its role in regulating human health. Numerous *Streptococcus* species are involved in carbohydrate fermentation, starch hydrolysis and glucan production from sucrose (Quan et al., 2019). *Streptococcus gallolyticus* can ferment mannitol, trehalose, and inulin and produce acids from starch and glycogen. Therefore, the presence of these bacteria suggest that the MF-TG pigs were healthier than the WT pigs because they have more probiotics to promote gut health or degrade carbohydrates in their diet. By contrast, the levels of *Campylobacter* and *Chlamydia* in the WT pigs were higher than those in the MF-TG pigs. *Campylobacter* and *Chlamydia* are common pathogenic bacterium in the digestive tract of numerous livestock, such as cattle, sheep, pig, and poultry, and often cause diarrhea and enteritis (Sachse and Grossmann, 2002; Lu et al., 2012; Burnham and Hendrixson, 2018). The presence of these pathogenic bacteria indicated that the WT pigs were more susceptible to diarrhea and enteritis than the MF-TG pigs. Other studies also suggested that the levels of NSP-degrading enzymes tend to increase the population of beneficial bacteria, thereby enhancing gut physiology, as evidenced by reducing relative weight of organs in the digestive system and increasing villus height (Zijlstra et al., 2010).

We also compared the functions and abundance of microbial genes of the MF-TG and WT pigs. Results showed that the abundance of K07258, K00610 and K06142 in the cecum of the MF-TG pigs was more active than that of WT pigs. These genes are associated with cysteine and methionine metabolism, peptidoglycan biosynthesis and nucleotide metabolism. Prior studies reported that pigs with high feed utilization have high abundance of methionine metabolism, peptidoglycan biosynthesis and nucleotide metabolism pathways. These features seem to verify that the gut microorganisms in the MF-TG pigs can adapt to multiple digestive enzymes and evolve new mechanisms to proliferate despite altered metabolic conditions (Quan et al., 2019). In addition, the relative abundances of carbohydrate enzymes involved in the hydrolysis of NSPs, starch, limit dextrins, fructans, sucroses, and trehaloses, and many phosphorylases related to carbohydrate metabolism in the cecal microbes of MF-TG pigs were high, which may be due to the high concentration of phosphates and oligosaccharides in the intestinal tract of the MF-TG pigs. These results on the functions of microbial genes indicated that the microorganisms promoted the adaptability of transgenic enzymes and increased the feed efficiency of the MF-TG pigs.

Moreover, our results showed that common veterinary drugs were associated with their corresponding resistance genes in the pig intestine. During the experiment, all of the pigs were not treated with any antibiotics. We speculate that the gut microorganisms carrying antibiotic resistance genes were obtained from the external environment where antibiotics resistant microorganisms exist pervasively because of the long history of antibiotics abuse. Nevertheless, the relative abundance and composition of the antimicrobial resistance genes were not significantly different between the MF-TG and WT pigs, suggesting that the development of antibiotic resistance has no relationship to the expression of exogenous digestive enzymes in the MF-TG pigs.

## Conclusion

The MF-TG pigs secreting NSP-degrading enzymes and phytase in the salivary glands can greatly promote nutrient absorption, improve growth performance and reduce pollutant emissions. Moreover, the intestinal microbiome exhibited adaptive changes to the transgenic enzymes, which may be beneficial to animal nutrient utilization and health.

## Data Availability Statement

The original contributions presented in the study are included in the article/Supplementary Material, further inquiries can be directed to the corresponding authors.

## Ethics Statement

The animal study was reviewed and approved by the Animal Care and Use Committee of the South China Agricultural University.

## Author Contributions

JM conducted the animal work, performed most of the laboratory work, and revised the manuscript. GL conducted part of the laboratory work and wrote the manuscript. GH, HW, JS, RZ, and GC helped to conduct the animal trial and part of the laboratory work. ZW and XZ designed the experiment, oversaw the development of the study, and wrote the last version of the manuscript. All authors contributed to the article and approved the submitted version.

## Conflict of Interest

JM, HW, JS, RZ, GC, ZW, and XZ were employed by company Wens Foodstuff Group Co., Ltd. The remaining authors declare that the research was conducted in the absence of any commercial or financial relationships that could be construed as a potential conflict of interest.
