# Supplementary material for: Adaptation of Gut Microbiome to Transgenic Pigs Secreting β-Glucanase, Xylanase, and Phytase
Source: Front Genet. 2021 Mar 4;12:631071. doi: 10.3389/fgene.2021.631071 (PMC7971306; doi:10.3389/fgene.2021.631071)
Supplement: Supplementary file 1 [file Data_Sheet_1.zip › Supplementary Table 12.docx]

**Supplementary Table 12** **Comparison of the feed efﬁciency between transgenic** **pigs (TG) fed experimental diets, their WT littermates (WT1)** **fed experimental diets, and the WT2 pigs fed** **commercial diets**

| Items | Boars^1^ | | | | | | Gilts^2^ | | | | | |
| --- | --- | --- | --- | --- | --- | --- | --- | --- | --- | --- | --- | --- |
|  | TG | WT1 | WT2 | *P* values | Change, % | | TG | WT1 | WT2 | *P* values | Change, % | |
|  |  |  |  |  | TG vs. WT1 | TG vs. WT2 |  |  |  |  | TG vs WT1 | TG vs WT2 |
| ADG, g/d | 976.69 | 862.51 | 955.98 | 0.157 | +13.21 |  | 1001.18^a^ | 831.08^b^ | 848.76^b^ | 0.0013 | +20.47 | +17.96 |
| FCR | 2.11^a^ | 2.43^b^ | 2.12^ab^ | 0.0027 | -13.1 |  | 2.24^a^ | 2.42^ab^ | 2.49^b^ | 0.02 | -7.43 | -10.04 |

^1^Transgenic boars (TG, 13 heads) and their WT littermates (WT1, 10 heads) fed experimental diets with low nitrogen, low digestive energy diets, and without supplemental phosphorus, WT2 boars fed commercial diets (11 heads).

^2^Transgenic gilts (TG, 5 heads) and their WT littermates (WT1, 6 heads) fed the same experimental diets as TG boars,WT2 gilts fed commercial diets (11，heads).

a,b,c Values in the same row with different superscript letters differ significantly (*P*<0.05).

ADG and FCR were recorded and analyzed from 50kg to 115kg.
