# Supplementary material for: Adaptation of Gut Microbiome to Transgenic Pigs Secreting β-Glucanase, Xylanase, and Phytase
Source: Front Genet. 2021 Mar 4;12:631071. doi: 10.3389/fgene.2021.631071 (PMC7971306; doi:10.3389/fgene.2021.631071)
Supplement: Supplementary file 1 [file Data_Sheet_1.zip › Supplementary Table 13.docx]

**Supplementary Table 13 Cloning information of marker-free transgenic (MF-TG) porcine fetal fibroblasts derived from a single colony.**

| No. of transferred embryos | No. of recipients | No. of farrowed recipients | No. of piglets | No. of piglets born alive | No. of stillborn piglets | Efficiency of clone |
| --- | --- | --- | --- | --- | --- | --- |
| 2001 | 8 | 4 | 19 | 17 | 2 | 0.85%(17/2001) |
