# Supplementary material for: Adaptation of Gut Microbiome to Transgenic Pigs Secreting β-Glucanase, Xylanase, and Phytase
Source: Front Genet. 2021 Mar 4;12:631071. doi: 10.3389/fgene.2021.631071 (PMC7971306; doi:10.3389/fgene.2021.631071)
Supplement: Supplementary file 1 [file Data_Sheet_1.zip › Supplementary Tables.docx]

**Supplementary Table 1**. **The primers information used for PCR amplification and southern blotting**

| Items | Sequence (5′-3′) | Annealing temperature (℃) | Deletion | |
| --- | --- | --- | --- | --- |
|  |  |  | Before | After |
| P1 | F:GGATACTTTATTATTCTCTGACTCGGTC | 57 | 1817 | 1817 |
|  | R:CATAGTTGGTTGAAGGAATGTGTGC |  |  |  |
| P2 | F:CTTTCACAGTGGTCACCCAGTTTC | 57 | 1004 | 1004 |
|  | R:TCAAAGGCATAGGTATGGTAAGCG |  |  |  |
| P3 | F:CATACGATGTTCCAGATTACGCTTG | 59 | 3643 | 747 |
|  | R:GAAGGACACACTATGGATGGGAGAC |  |  |  |
| Probe 1 | F:CTTTCACAGTGGTCACCCAGTTTC | 57 | 1004 | 1004 |
|  | R:TCAAAGGCATAGGTATGGTAAGCG |  |  |  |
| Probe 2 | F:CGCTTTTCTGGATTCATCGAC | 56 | 917 | 0 |
|  | R:CTAGATTACTTGTACAGCTCGT |  |  |  |

**Supplementary Table 2 Generation of marker-free transgenic offsprings**

| Fathers | Mothers | Number | | |
| --- | --- | --- | --- | --- |
|  |  | Piglets | TG | WT |
| 907 | AB16-339900 | 5 | 2 | 3 |
|  | AB15-568406 | 5 | 2 | 3 |
|  | AB15-128300 | 6 | 4 | 2 |
|  | AB15-569514 | 4 | 3 | 1 |
| 903 | AB15-568606 | 4 | 2 | 2 |
|  | AB16-300500 | 6 | 3 | 3 |
| 803 | AB16-342502 | 6 | 3 | 3 |
|  | AB15-581106 | 5 | 3 | 2 |
|  | AB15-103700 | 7 | 2 | 5 |
| Total | 9 | 48 | 24 | 24 |

mothers were WT Duroc sows.

**Supplementary Table 3 Distribution of MK3 FIRE** **feeders for** **measuring the growth performance of transgenic pigs**

| Feeders | Pigs | Number |
| --- | --- | --- |
| #1 | TG♀ | 6 |
| #2 | WT♀ | 6 |
| #3 | TG♀+WT♀ | 11 (5TG, 5WT) |
| #4 | TG♂ | 13 |
| #5 | WT♂ | 11 |
| Total |  | 46 |

**Supplementary Table 4 Ingredients and nutrient composition of daily diets for examining the efficiency of nutrient utilization in different grower pigs (30 – 115 kg)**

| Items | Grower pigs | | |
| --- | --- | --- | --- |
|  | 30~60kg | 60~90kg | 90~115kg |
| Corn | 361.50 | 260.00 | 240.00 |
| Wheat | 235.00 | 311.00 | 220.00 |
| Barley | 129.00 | 175.00 | 200.00 |
| Rice bran meal | 50.00 | 32.00 | - |
| Wheat bran | - | 20.00 | 162.00 |
| Soybean | 149.00 | 135.00 | 96.00 |
| Soybean meal (46 % crude protein) | 15.00 | - | - |
| Soybean oil | 20.00 | 31.00 | 50.00 |
| Sodium chloride | 16.60 | 12.00 | 11.00 |
| Premix^1^ | 10.00 |  |  |
| Premix^2^ |  | 10 | 10 |
| L-Lysine sulphate, 70% | 5.30 | 5.60 | 3.50 |
| L-Threonine, 98.5% | 2.10 | 1.70 | 0.90 |
| L-Methionine, 98% | 1.00 | 0.80 | 0.40 |
| Tryptophan, 98% | 0.50 | 0.40 | 0.20 |
| Sodium chloride | 3.00 | 3.00 | 3.00 |
| Choline chloride, 60% | 1.00 | 1.50 | 2.00 |
| Mildewcide | 1.00 | 1.00 | 1.00 |
| Total, kg | 1000.00 | 1000.00 | 1000.00 |
| Digestible energy (DE), Kcal/kg | 3339 | 3294 | 3209 |
| Crude protein (CP),% | 14.94 | 13.9 | 12.96 |
| Total calcium (Ca),% | 0.69 | 0.62 | 0.63 |
| Total phosphorus (Pi), % | 0.4 | 0.38 | 0.39 |
| Available phosphorus,% | 0.103 | 0.124 | 0.153 |

1. Provided by Wen's Food Group Co., Ltd., per kg of diet includes: Vitamin A 6500 IU, Vitamin D3 2000IU, Vitamin E 42.00mg, Vitamin K3 2.00mg, Vitamin B1 2.00mg, Vitamin B2 6.40mg, vitamin B6 3.00mg, vitamin B12 0.02mg, D-biotin 0.16mg, D-pantothenic acid 20.00mg, folic acid 1.20 mg, niacin 24.00 mg, Fe 159.00 mg, Zn 161.00 mg, Cu142.00mg, Mg 40.00mg, I 0.40 mg, Se 0.30 mg, Co0.10 mg, 8% enalamycin 100 mg. Antioxidant 85 mg .;

2 Provided by Wen's Food Group Co., Ltd., each kg of diet includes: vitamin A, 6500 IU, vitamin D 2000 IU, vitamin E 40 mg, vitamin K3 2.00 mg, vitamin B1 2.00 mg, vitamin B2 5.00 mg, vitamin B2 6.40 mg, vitamin B6 3.00 mg, vitamin B12 0.02 mg, biotin 0.15 mg, D-pantothenic acid 20.00 mg, folic acid 1.00 mg, nicotinic acid 24.00 mg, Fe 134.00 mg, Zn125.00 mg, Cu 30.00 mg, Mg50.00 mg, I 0.50 mg, Se 0.35 mg, Co 0.16 mg; 8% enalamycin 100 mg. Antioxidant 85 mg.

**Supplementary Table 5 Number of microorganisms that can be annotated into the NR database.**

| Items | Annotation on different | |
| --- | --- | --- |
|  | Genes number | Percentage (%) |
| Gene catalogue | 2,571,884 |  |
| NR database | 1,832,628 | 71.26 |
| Unclassified | 314,296 | 17.15 |
| Kingdom | 1,518,332 | 82.85 |
| Phylum | 1,448,509 | 79.04 |
| Class | 1,334,153 | 72.80 |
| Order | 1,324,074 | 72.25 |
| Family | 11,008,606 | 60.07 |
| Genus | 10,119,776 | 55.22 |
| Species | 779,417 | 42.53 |

**Supplementary Table 7 Based on the abundance of phylum level, principal component analysis (PCA) were analyzed between different groups**

|  | Dim.1 | Dim.2 | Dim.3 | Dim.4 | Dim.5 |
| --- | --- | --- | --- | --- | --- |
| Ce.T1F | -1.50287 | -0.03739 | -0.83245 | -0.67381 | -2.64969 |
| Ce.T2F | 3.946345 | 0.457299 | -0.46705 | -1.0837 | -1.2787 |
| Ce.T3F | -0.77848 | 1.317182 | 0.526411 | -1.16159 | -2.19297 |
| Ce.T4F | -1.66367 | 0.675991 | -0.65093 | -0.94633 | -2.32395 |
| Ce.T5F | 9.273401 | 4.877824 | -0.25493 | 1.946791 | 1.306515 |
| Ce.W1F | 7.480843 | 6.733635 | 1.0372 | -3.61797 | -0.45502 |
| Ce.W2F | 7.510311 | 10.89771 | 4.852917 | -8.50693 | -2.65524 |
| Ce.W3F | 4.661903 | 3.463141 | -0.78524 | -3.25546 | -0.00262 |
| Ce.W4F | 6.788617 | 3.08467 | -0.29403 | -2.69417 | 0.385321 |
| Ce.W5F | 4.621837 | 0.545326 | -1.47024 | 1.364327 | 1.205699 |
| Co.T1F | 2.007094 | -0.98116 | -0.96491 | 0.117072 | -1.16644 |
| Co.T2F | 2.217351 | -2.71787 | -1.07655 | 0.491486 | -1.66414 |
| Co.T3F | 0.561586 | 0.293859 | 0.750416 | -0.44427 | -1.34683 |
| Co.T4F | 1.860246 | -0.77647 | -0.01008 | -1.37421 | -2.12722 |
| Co.T5F | 1.264936 | -2.53516 | -0.90424 | -0.14544 | -1.09953 |
| Co.W1F | 9.627394 | -9.12021 | 2.868853 | -0.18164 | 2.168089 |
| Co.W2F | 3.383851 | -0.2289 | 0.336963 | -1.88265 | -1.61519 |
| Co.W3F | 10.34568 | -11.6677 | 9.97979 | -3.54843 | 5.730864 |
| Co.W4F | 6.845427 | -3.37547 | -1.55449 | -1.66 | 2.005733 |
| Co.W5F | 2.545974 | -2.34119 | -0.48403 | 0.921407 | 0.109384 |
| IL.T1F | -4.94196 | 2.647871 | 0.478667 | -1.00109 | -1.96486 |
| IL.T2F | -6.41675 | -0.26652 | -0.22765 | -0.36371 | -0.3289 |
| IL.T3F | -8.76628 | 0.699868 | -0.9968 | -1.70948 | 5.107913 |
| IL.T4F | -7.49718 | -0.92852 | -0.73041 | -2.04755 | 4.065359 |
| IL.T5F | -6.32277 | 0.795619 | -0.53726 | -1.13999 | 1.724885 |
| IL.W1F | -6.23676 | 1.04126 | 1.228319 | 0.304788 | 0.303987 |
| IL.W2F | 2.843037 | 5.613038 | 1.92711 | -4.8572 | -2.87202 |
| IL.W3F | -6.98607 | 0.404802 | -0.42491 | -0.56793 | 2.332919 |
| IL.W4F | -8.71271 | 0.712646 | -1.19118 | -1.21082 | 5.771558 |
| IL.W5F | -7.93415 | 0.830747 | -0.19465 | 0.004887 | 3.332937 |

**Supplementary Table 8 Number of ARO was no significant difference between TG pigs and WT pigs**

| Items | Average number | | | | | |
| --- | --- | --- | --- | --- | --- | --- |
|  |  | 1 | 2 | 3 | 4 | 5 |
| Ce | TG | 302 | 330 | 283 | 294 | 311 |
|  | WT | 317 | 291 | 276 | 273 | 272 |
| Co | TG | 331 | 340 | 304 | 322 | 311 |
|  | WT | 328 | 318 | 296 | 283 | 301 |
| IL | TG | 88 | 213 | 31 | 78 | 108 |
|  | WT | 93 | 304 | 72 | 41 | 78 |

**Supplementary Table 10 The significantly enriched carbohydrases in the caecum of MF-TG pigs.**

| \| EC_ID \| Ce.T1F \| Ce.T2F \| Ce.T3F \| Ce.T4F \| Ce.W1F \| Ce.W2F \| Ce.W3F \| Ce.W4F \| Ce.W5F \| \| --- \| --- \| --- \| --- \| --- \| --- \| --- \| --- \| --- \| --- \| \| glucan 1,3-beta-glucosidase (EC 3.2.1.58) \| 0.001212292 \| 0.000953 \| 0.000966654 \| 0.001007223 \| 0.000578994 \| 0.000467429 \| 0.00079869 \| 0.000635762 \| 0.000690844 \| \| beta-N-acetylglucosaminide phosphorylases (EC 2.4.1.-) \| 0.001210319 \| 0.000952785 \| 0.000966042 \| 0.00100353 \| 0.000577937 \| 0.000466796 \| 0.000798307 \| 0.000635335 \| 0.000688169 \| \| beta-N-acetylhexosaminidase (EC 3.2.1.52) \| 0.001210319 \| 0.000952785 \| 0.000966042 \| 0.00100353 \| 0.000577937 \| 0.000466796 \| 0.000798307 \| 0.000635335 \| 0.000688169 \| \| coniferin beta-glucosidase (EC 3.2.1.126) \| 0.001210319 \| 0.000952785 \| 0.000966042 \| 0.00100353 \| 0.000577937 \| 0.000466796 \| 0.000798307 \| 0.000635335 \| 0.000688169 \| \| exo-1,3-1,4-glucanase (EC 3.2.1.-) \| 0.001210319 \| 0.000952785 \| 0.000966042 \| 0.00100353 \| 0.000577937 \| 0.000466796 \| 0.000798307 \| 0.000635335 \| 0.000688169 \| \| glucan 1,4-beta-glucosidase (EC 3.2.1.74) \| 0.001210319 \| 0.000952785 \| 0.000966042 \| 0.00100353 \| 0.000577937 \| 0.000466796 \| 0.000798307 \| 0.000635335 \| 0.000688169 \| \| xylan 1,4-beta-xylosidase (EC 3.2.1.37) \| 0.001210319 \| 0.000952785 \| 0.000966042 \| 0.00100353 \| 0.000577937 \| 0.000466796 \| 0.000798307 \| 0.000635335 \| 0.000688169 \| \| 4-alpha-glucanotransferase (EC 2.4.1.25) \| 0.001258596 \| 0.001005546 \| 0.001248159 \| 0.001126847 \| 0.000749769 \| 0.000783761 \| 0.000932527 \| 0.000807212 \| 0.000880921 \| \| alpha-amylase (EC 3.2.1.1) \| 0.001258596 \| 0.001005546 \| 0.001248159 \| 0.001126847 \| 0.000749769 \| 0.000783761 \| 0.000932527 \| 0.000807212 \| 0.000880921 \| \| branching enzyme (EC 2.4.1.18) \| 0.001258596 \| 0.001005546 \| 0.001248159 \| 0.001126847 \| 0.000749769 \| 0.000783761 \| 0.000932527 \| 0.000807212 \| 0.000880921 \| \| cyclomaltodextrinase (EC 3.2.1.54) \| 0.001258596 \| 0.001005546 \| 0.001248159 \| 0.001126847 \| 0.000749769 \| 0.000783761 \| 0.000932527 \| 0.000807212 \| 0.000880921 \| \| glucodextranase (EC 3.2.1.70) \| 0.001232998 \| 0.000964958 \| 0.001201772 \| 0.001085285 \| 0.000728295 \| 0.000759963 \| 0.000895856 \| 0.000769428 \| 0.000837023 \| \| 6_-P-sucrose phosphorylase (EC 2.4.1.-) \| 0.001232859 \| 0.000964958 \| 0.001201242 \| 0.001085285 \| 0.000727813 \| 0.000759772 \| 0.000895856 \| 0.000769428 \| 0.000837023 \| \| amylosucrase (EC 2.4.1.4) \| 0.001232859 \| 0.000964958 \| 0.001201242 \| 0.001085285 \| 0.000727813 \| 0.000759772 \| 0.000895856 \| 0.000769428 \| 0.000837023 \| \| cyclomaltodextrin glucanotransferase (EC 2.4.1.19) \| 0.001232859 \| 0.000964958 \| 0.001201242 \| 0.001085285 \| 0.000727813 \| 0.000759772 \| 0.000895856 \| 0.000769428 \| 0.000837023 \| \| isoamylase (EC 3.2.1.68) \| 0.001232859 \| 0.000964958 \| 0.001201242 \| 0.001085285 \| 0.000727813 \| 0.000759772 \| 0.000895856 \| 0.000769428 \| 0.000837023 \| \| isomaltulose synthase (EC 5.4.99.11) \| 0.001232859 \| 0.000964958 \| 0.001201242 \| 0.001085285 \| 0.000727813 \| 0.000759772 \| 0.000895856 \| 0.000769428 \| 0.000837023 \| \| malto-oligosyltrehalose synthase (EC 5.4.99.15) \| 0.001232859 \| 0.000964958 \| 0.001201242 \| 0.001085285 \| 0.000727813 \| 0.000759772 \| 0.000895856 \| 0.000769428 \| 0.000837023 \| \| malto-oligosyltrehalose trehalohydrolase (EC 3.2.1.141) \| 0.001232859 \| 0.000964958 \| 0.001201242 \| 0.001085285 \| 0.000727813 \| 0.000759772 \| 0.000895856 \| 0.000769428 \| 0.000837023 \| \| maltogenic amylase (EC 3.2.1.133) \| 0.001232859 \| 0.000964958 \| 0.001201242 \| 0.001085285 \| 0.000727813 \| 0.000759772 \| 0.000895856 \| 0.000769428 \| 0.000837023 \| \| maltohexaose-forming alpha-amylase (EC 3.2.1.98) \| 0.001232859 \| 0.000964958 \| 0.001201242 \| 0.001085285 \| 0.000727813 \| 0.000759772 \| 0.000895856 \| 0.000769428 \| 0.000837023 \| \| maltopentaose-forming alpha-amylase (EC 3.2.1.-) \| 0.001232859 \| 0.000964958 \| 0.001201242 \| 0.001085285 \| 0.000727813 \| 0.000759772 \| 0.000895856 \| 0.000769428 \| 0.000837023 \| \| maltotetraose-forming alpha-amylase (EC 3.2.1.60) \| 0.001232859 \| 0.000964958 \| 0.001201242 \| 0.001085285 \| 0.000727813 \| 0.000759772 \| 0.000895856 \| 0.000769428 \| 0.000837023 \| \| maltotriose-forming alpha-amylase (EC 3.2.1.116) \| 0.001232859 \| 0.000964958 \| 0.001201242 \| 0.001085285 \| 0.000727813 \| 0.000759772 \| 0.000895856 \| 0.000769428 \| 0.000837023 \| \| neopullulanase (EC 3.2.1.135) \| 0.001232859 \| 0.000964958 \| 0.001201242 \| 0.001085285 \| 0.000727813 \| 0.000759772 \| 0.000895856 \| 0.000769428 \| 0.000837023 \| \| oligo-alpha-glucosidase (EC 3.2.1.10) \| 0.001232859 \| 0.000964958 \| 0.001201242 \| 0.001085285 \| 0.000727813 \| 0.000759772 \| 0.000895856 \| 0.000769428 \| 0.000837023 \| \| pullulanase (EC 3.2.1.41) \| 0.001232859 \| 0.000964958 \| 0.001201242 \| 0.001085285 \| 0.000727813 \| 0.000759772 \| 0.000895856 \| 0.000769428 \| 0.000837023 \| \| sucrose phosphorylase (EC 2.4.1.7) \| 0.001232859 \| 0.000964958 \| 0.001201242 \| 0.001085285 \| 0.000727813 \| 0.000759772 \| 0.000895856 \| 0.000769428 \| 0.000837023 \| \| trehalose synthase (EC 5.4.99.16) \| 0.001232859 \| 0.000964958 \| 0.001201242 \| 0.001085285 \| 0.000727813 \| 0.000759772 \| 0.000895856 \| 0.000769428 \| 0.000837023 \| \| trehalose-6-phosphate hydrolase (EC 3.2.1.93) \| 0.001232859 \| 0.000964958 \| 0.001201242 \| 0.001085285 \| 0.000727813 \| 0.000759772 \| 0.000895856 \| 0.000769428 \| 0.000837023 \| \| beta-2,6-fructan 6-levanbiohydrolase (EC 3.2.1.64) \| 0.000470357 \| 0.000398053 \| 0.000418561 \| 0.000408471 \| 0.000279472 \| 0.000339335 \| 0.00030631 \| 0.000293333 \| 0.000224278 \| \| cycloinulo-oligosaccharide fructanotransferase (EC 2.4.1.-) \| 0.000470357 \| 0.000398053 \| 0.000418561 \| 0.000408471 \| 0.000279472 \| 0.000339335 \| 0.00030631 \| 0.000293333 \| 0.000224278 \| \| endo-inulinase (EC 3.2.1.7) \| 0.000470357 \| 0.000398053 \| 0.000418561 \| 0.000408471 \| 0.000279472 \| 0.000339335 \| 0.00030631 \| 0.000293333 \| 0.000224278 \| \| endo-levanase (EC 3.2.1.65) \| 0.000470357 \| 0.000398053 \| 0.000418561 \| 0.000408471 \| 0.000279472 \| 0.000339335 \| 0.00030631 \| 0.000293333 \| 0.000224278 \| \| exo-inulinase (EC 3.2.1.80) \| 0.000470357 \| 0.000398053 \| 0.000418561 \| 0.000408471 \| 0.000279472 \| 0.000339335 \| 0.00030631 \| 0.000293333 \| 0.000224278 \| \| fructan:fructan 1-fructosyltransferase (EC 2.4.1.100) \| 0.000470357 \| 0.000398053 \| 0.000418561 \| 0.000408471 \| 0.000279472 \| 0.000339335 \| 0.00030631 \| 0.000293333 \| 0.000224278 \| \| fructan:fructan 6G-fructosyltransferase (EC 2.4.1.243) \| 0.000470357 \| 0.000398053 \| 0.000418561 \| 0.000408471 \| 0.000279472 \| 0.000339335 \| 0.00030631 \| 0.000293333 \| 0.000224278 \| \| invertase (EC 3.2.1.26) \| 0.000470357 \| 0.000398053 \| 0.000418561 \| 0.000408471 \| 0.000279472 \| 0.000339335 \| 0.00030631 \| 0.000293333 \| 0.000224278 \| \| levan fructosyltransferase (EC 2.4.1.-) \| 0.000470357 \| 0.000398053 \| 0.000418561 \| 0.000408471 \| 0.000279472 \| 0.000339335 \| 0.00030631 \| 0.000293333 \| 0.000224278 \| \| sucrose:fructan 6-fructosyltransferase (EC 2.4.1.10) \| 0.000470357 \| 0.000398053 \| 0.000418561 \| 0.000408471 \| 0.000279472 \| 0.000339335 \| 0.00030631 \| 0.000293333 \| 0.000224278 \| \| sucrose:sucrose 1-fructosyltransferase (EC 2.4.1.99) \| 0.000470357 \| 0.000398053 \| 0.000418561 \| 0.000408471 \| 0.000279472 \| 0.000339335 \| 0.00030631 \| 0.000293333 \| 0.000224278 \| \| beta-1,2-oligoglucan phosphorylase (EC 2.4.1.-) \| 0.000266355 \| 0.000254783 \| 0.000254552 \| 0.00019303 \| 0.000148345 \| 0.000138413 \| 0.000121012 \| 0.000115716 \| 0.000131331 \| \| cellobionic acid phosphorylase (EC 2.4.1.321) \| 0.000266355 \| 0.000254783 \| 0.000254552 \| 0.00019303 \| 0.000148345 \| 0.000138413 \| 0.000121012 \| 0.000115716 \| 0.000131331 \| \| cellobiose phosphorylase (EC 2.4.1.20) \| 0.000266355 \| 0.000254783 \| 0.000254552 \| 0.00019303 \| 0.000148345 \| 0.000138413 \| 0.000121012 \| 0.000115716 \| 0.000131331 \| \| cellodextrin phosphorylase (EC 2.4.1.49) \| 0.000266355 \| 0.000254783 \| 0.000254552 \| 0.00019303 \| 0.000148345 \| 0.000138413 \| 0.000121012 \| 0.000115716 \| 0.000131331 \| \| chitobiose phosphorylase (EC 2.4.1.-) \| 0.000266355 \| 0.000254783 \| 0.000254552 \| 0.00019303 \| 0.000148345 \| 0.000138413 \| 0.000121012 \| 0.000115716 \| 0.000131331 \| \| cyclic beta-1,2-glucan synthase (EC 2.4.1.-) \| 0.00026669 \| 0.000255114 \| 0.000255516 \| 0.000193096 \| 0.000148345 \| 0.000138413 \| 0.000121185 \| 0.000115716 \| 0.000131331 \| \| laminaribiose phosphorylase (EC 2.4.1.31) \| 0.000266355 \| 0.000254783 \| 0.000254552 \| 0.00019303 \| 0.000148345 \| 0.000138413 \| 0.000121012 \| 0.000115716 \| 0.000131331 \| \| alpha-1,3-L-neoagarooligosaccharide hydrolase (EC 3.2.1.-) \| 3.02E-06 \| 2.31E-06 \| 1.69E-06 \| 1.81E-06 \| 6.77E-07 \| 1.63E-07 \| 1.30E-06 \| 9.32E-07 \| 1.86E-06 \| \| beta-1,3-N-acetylglucosaminyltransferase (EC 2.4.1.-) \| 3.95E-07 \| 3.88E-07 \| 0 \| 4.44E-07 \| 0 \| 0 \| 9.20E-08 \| 0 \| 9.53E-08 \| |
| --- | --- | --- | --- | --- | --- | --- | --- | --- | --- | --- | --- | --- | --- | --- | --- | --- | --- | --- | --- | --- | --- | --- | --- | --- | --- | --- | --- | --- | --- | --- | --- | --- | --- | --- | --- | --- | --- | --- | --- | --- | --- | --- | --- | --- | --- | --- | --- | --- | --- | --- | --- | --- | --- | --- | --- | --- | --- | --- | --- | --- | --- | --- | --- | --- | --- | --- | --- | --- | --- | --- | --- | --- | --- | --- | --- | --- | --- | --- | --- | --- | --- | --- | --- | --- | --- | --- | --- | --- | --- | --- | --- | --- | --- | --- | --- | --- | --- | --- | --- | --- | --- | --- | --- | --- | --- | --- | --- | --- | --- | --- | --- | --- | --- | --- | --- | --- | --- | --- | --- | --- | --- | --- | --- | --- | --- | --- | --- | --- | --- | --- | --- | --- | --- | --- | --- | --- | --- | --- | --- | --- | --- | --- | --- | --- | --- | --- | --- | --- | --- | --- | --- | --- | --- | --- | --- | --- | --- | --- | --- | --- | --- | --- | --- | --- | --- | --- | --- | --- | --- | --- | --- | --- | --- | --- | --- | --- | --- | --- | --- | --- | --- | --- | --- | --- | --- | --- | --- | --- | --- | --- | --- | --- | --- | --- | --- | --- | --- | --- | --- | --- | --- | --- | --- | --- | --- | --- | --- | --- | --- | --- | --- | --- | --- | --- | --- | --- | --- | --- | --- | --- | --- | --- | --- | --- | --- | --- | --- | --- | --- | --- | --- | --- | --- | --- | --- | --- | --- | --- | --- | --- | --- | --- | --- | --- | --- | --- | --- | --- | --- | --- | --- | --- | --- | --- | --- | --- | --- | --- | --- | --- | --- | --- | --- | --- | --- | --- | --- | --- | --- | --- | --- | --- | --- | --- | --- | --- | --- | --- | --- | --- | --- | --- | --- | --- | --- | --- | --- | --- | --- | --- | --- | --- | --- | --- | --- | --- | --- | --- | --- | --- | --- | --- | --- | --- | --- | --- | --- | --- | --- | --- | --- | --- | --- | --- | --- | --- | --- | --- | --- | --- | --- | --- | --- | --- | --- | --- | --- | --- | --- | --- | --- | --- | --- | --- | --- | --- | --- | --- | --- | --- | --- | --- | --- | --- | --- | --- | --- | --- | --- | --- | --- | --- | --- | --- | --- | --- | --- | --- | --- | --- | --- | --- | --- | --- | --- | --- | --- | --- | --- | --- | --- | --- | --- | --- | --- | --- | --- | --- | --- | --- | --- | --- | --- | --- | --- | --- | --- | --- | --- | --- | --- | --- | --- | --- | --- | --- | --- | --- | --- | --- | --- | --- | --- | --- | --- | --- | --- | --- | --- | --- | --- | --- | --- | --- | --- | --- | --- | --- | --- | --- | --- | --- | --- | --- | --- | --- | --- | --- | --- | --- | --- | --- | --- | --- | --- | --- | --- | --- | --- | --- | --- | --- | --- | --- | --- | --- | --- | --- | --- | --- | --- | --- | --- | --- | --- | --- | --- | --- | --- | --- | --- | --- | --- | --- | --- | --- | --- | --- | --- | --- | --- | --- | --- | --- | --- | --- | --- | --- | --- | --- | --- | --- | --- | --- | --- | --- | --- | --- | --- | --- | --- | --- | --- | --- | --- | --- | --- | --- | --- | --- | --- | --- | --- | --- | --- | --- | --- | --- | --- | --- |

**Supplementary Table 11 Major nutrient content in the commercial formula feed for breeding pigs**

| Nutrient | Unit | stage | | |
| --- | --- | --- | --- | --- |
|  |  | <50kg | 50-100kg | 100-130kg |
| Protein | % | 17.5 | 16.0 | 15.0 |
| calcium | % | 0.95 | 0.90 | 0.90 |
| Apparent digestible phosphorus | % | 0.30 | 0.27 | 0.25 |
| net energy | kcal/kg | 2450 | 2420 | 2320 |
